# The Use of UV-Visible Diffuse Reflectance Spectrophotometry for a Fast, Preliminary Authentication of Gemstones

**DOI:** 10.3390/molecules27154716

**Published:** 2022-07-23

**Authors:** Maurizio Aceto, Elisa Calà, Federica Gulino, Francesca Gullo, Maria Labate, Angelo Agostino, Marcello Picollo

**Affiliations:** 1 Dipartimento per lo Sviluppo Sostenibile e la Transizione Ecologica (DiSSTE), Università degli Studi del Piemonte Orientale, Piazza Sant’Eusebio, 5-13100 Vercelli, Italy; elisa.cala@uniupo.it (E.C.); federica.gulino@uniupo.it (F.G.); francesca.gullo@uniupo.it (F.G.); 2 Dipartimento di Chimica, Università degli Studi di Torino, Via P. Giuria, 7-10125 Torino, Italy; maria.labate@unito.it (M.L.); angelo.agostino@unito.it (A.A.); 3 Istituto di Fisica Applicata “Nello Carrara” del Consiglio Nazionale delle Ricerche (IFAC-CNR), Via Madonna del Piano, 10-50019 Sesto Fiorentino, Italy; m.picollo@ifac.cnr.it

**Keywords:** FORS, reflectance, non-invasive, gemstones, colour

## Abstract

The identification of gemstones is an important topic in the field of cultural heritage, given their enormous value. Particularly, the most important precious stones, namely diamond, emerald, ruby and sapphire, are frequently subjected to counterfeit by substitution with objects of lesser value with similar appearance, colour or shape. While a gemmologist is able to recognise a counterfeit in most instances, more generally, it is not easy to do this without resorting to instrumental methods. In this work, the use of UV-visible diffuse reflectance spectrophotometry with optic fibres (FORS) is proposed as a fast and easy method for the preliminary identification of gemstones, alternative to the classical methods used by gemmologists or to Raman spectroscopy, which is by far the instrumental method with the best diagnostic potential, but it cannot be used in situations of problematic geometric hindrance. The possibilities and the limitations given by the FORS technique are critically discussed together with the spectral features of the most important gemstones. Finally, the application of chemometric pattern recognition methods is described for the treatment of large sets of spectral data deriving from gemstones identification.

## 1. Introduction

Due to their enormous value, the identification of gemstones is an important topic in the field of cultural heritage. Counterfeit is rather common, and particularly, the most precious stones, namely diamond, emerald, ruby and sapphire, are frequently substituted with gemstones of lesser value which have the same appearance, colour or shape. While a skilful gemmologist with expertise may be able to differentiate between authentic and fake gemstones, it can be challenging without resorting to instrumental methods.

The classical methods used by gemmologists are based on the measurement of the refraction index, of the birefringence or double refraction and of specific gravity [1,2]. Apart from these methods, instrumental techniques are presently available which allow even non-experts to correctly identify gemstones. Raman spectroscopy is one of the techniques with the most accurate diagnostic potential due to the fact that it can provide a fingerprint of nearly every known gemstone [3,4]; moreover, the process is completely non-invasive and non-destructive. When a portable Raman system is available, this analysis can be performed in situ, i.e., inside museums where precious artworks are kept, without the need to remove the objects from their original location in order to analyse them in laboratories [5,6,7,8]. Fourier Transform-Infrared (FT-IR) spectroscopy can be used as well, exploiting the various configurations available, i.e., absorbance, reflectance, Attenuated Total Reflection (ATR), etc. [9].

Despite the reliability of Raman spectroscopy in the identification of gemstones, in certain situations, it cannot be used because of geometric hindrances (e.g., an artwork located inside a cabinet). The same holds when a jewellery artwork is to be analysed using classical methods that need gemstones to be studied detached from the jewel frame.

Another technique commonly used in gemmological laboratories is UV-Visible-NIR absorption spectrophotometry. It is well known that most gemstones owe their colour to the presence of small amounts of transition metal ions occurring as impurities inside their structure [10,11]: such gemstones are called *allochromatic*, in contrast to *idiochromatic* gemstones in which the chromophore is a main chemical constituent (an example is turquoise -CuAl_6_(PO_4_)_4_(OH)_8_·4H_2_O-in which the chromophore is Cu^2+^). Particularly relevant is the incomplete set of 3d electrons of transition metal ions. Since most of the d–d transitions occur in the visible region, UV-Visible-NIR absorption spectrophotometry is suitable for the identification of coloured gemstones. Other known phenomena causing the appearance of colour in gemstones, such as charge-transfer and colour centres, generate spectral features [12,13,14] that can be appreciated as well. However, this technique has two main drawbacks in the analysis of gemstones: (1) when used in absorbance mode, it functions on transparent gemstones only; (2) it can be used only on gemstones analysed in a laboratory. The first drawback can be addressed by means of an integration sphere, a sampling geometry that enables the collecting of reflectance spectra even from opaque or translucid gemstones, thus allowing to obtain *apparent absorbance* spectra; the second drawback cannot be addressed.

One additional drawback of UV-Visible-NIR absorption spectrophotometry is the fact that many gemstones are *pleochroic*, that is, they show two or three different colours when viewed from different angles or irradiated with different lights. This means that although such gemstones can be identified with Raman spectroscopy because the vibrational behaviour does not change even when the angle is changed, the absorption response—and therefore the possibility to reliably identify these gemstone—will vary because of both the different chromophore system present and the angle of collection of the response itself.

Finally, it is well known that artificial treatments, such as heating or irradiation, can cause colour changes due to induction (or improvement) of the charge transfer mechanism or creation of colour centres. In such cases, again, the absorption spectrum is changed while the vibrational spectrum is not.

In such cases, a good alternative could be the use of a preliminary technique such as UV-Visible diffuse reflectance spectrophotometry with optic fibres (FORS). The FORS technique, due to the use of a small probe, can be employed in situ nearly anywhere without steric constraints; moreover, the technique works on transparent, translucent and opaque objects. A patented method has been recently issued by Takahashi and Perera [15]. When examining pleochroic gemstones, it is relatively easy to change the angle of measurement in order to verify the different responses. The diagnostic issues generated by the artificial treatments of gemstones are obviously like those encountered in the absorption mode. Ultimately, the FORS technique can be an advantageous alternative to classical methods used in gemmology, in particular in cases where jewellery artworks cannot be moved from their natural locations, such as museums.

In this work, the possibilities and the limitations given by the FORS technique in the identification of gemstones are critically discussed.

## 2. Results

The spectral response yielded by the FORS analysis is mainly related to the chromophore system of the gemstone. In most cases, this involves the presence of one or more metal ions in specific oxidation states. The spectrum can therefore provide information useful for (a) the identification of the gemstone and (b) its geographical or geological provenance that can be related to the presence of specific elements.

The FORS technique is particularly useful in cases where the analysis is carried out on opaque material. In fact, these are the cases in which a larger amount of light is diffused by the sample because of scattering and therefore can reach the detection system. Most of the gemstones, however, are transparent or translucent; hence, the amount of reflected radiation is low and poor spectra must be expected. Nevertheless, the spectral features necessary for the identification (reflectance minima/apparent absorbance maxima) can usually be detected with instruments of good sensitivity.

**Figure 1:**
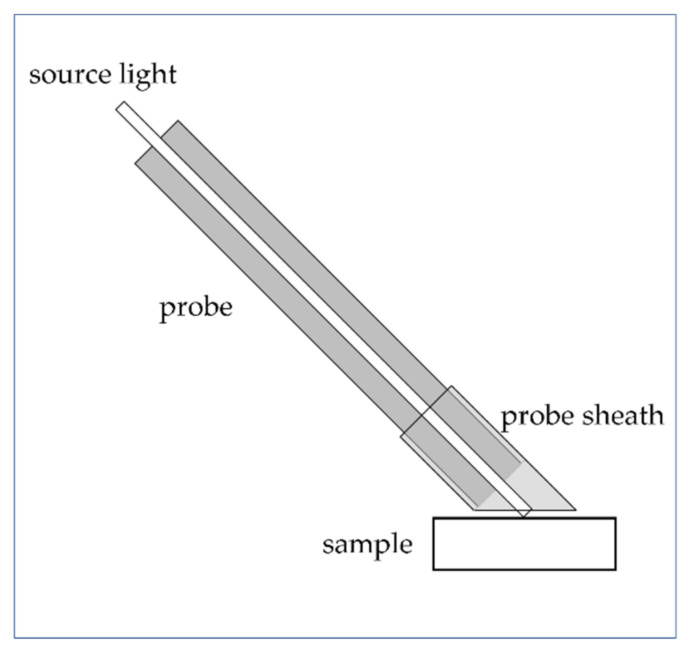
FORS probe with a small cylindrical sheath to exclude external source of light.

In FORS analysis of gemstones, the influence of ambient light (LED, neon lights, direct sunlight) on the spectral response must be taken into account. To exploit the advantages of this technique, the measurements are usually carried out in open systems, that is, presenting the probe directly to the gemstone without covers, contrarily to the measurements carried out inside spectrophotometers such as in transmission mode or with an integration sphere. Ambient light can generate undesired spectral artefacts, which are sometimes easy to recognise because they occur as sharp bands. A proper way to avoid this drawback is to cover the tip of the probe with a small cylindrical sheath cut into a slope (Figure 1) to exclude external sources of light.

In the following paragraphs, the possibility of identifying the most important gemstones with FORS will be evaluated. The discussion is arranged according to the colour of gemstones. Spectra are shown mostly in Log(1/R) coordinates, i.e., in *apparent absorbance*, in order to better appreciate the absorption features, except for cases in which luminescence bands must be highlighted (e.g., sapphire, ruby), and so, the corresponding spectra are shown in the usual reflectance coordinates. In the figures where multiple spectra are presented, spectra are offset for clarity.

The identification of all the gemstones analysed has been previously confirmed by means of Raman spectroscopy.

### 2.1. Blue Gemstones

**Figure 2:**
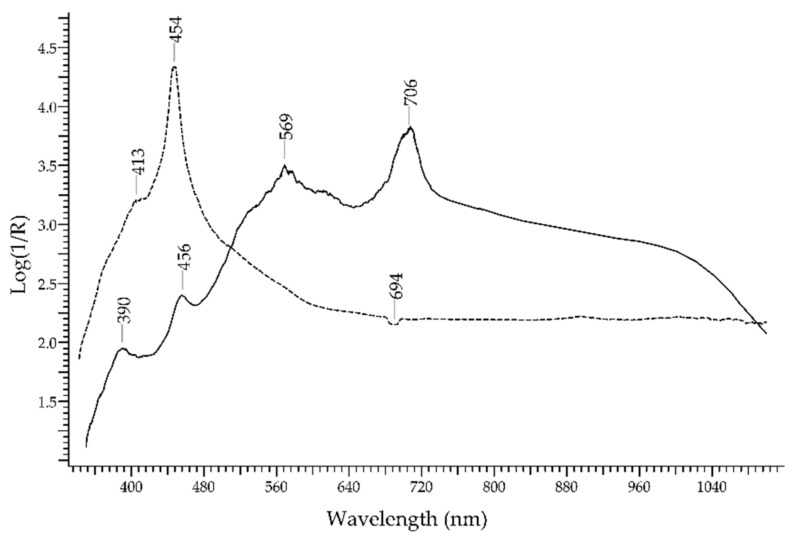
FORS spectrum in reflectance coordinates of a blue sapphire from Cambodia (solid line) and a yellow synthetic sapphire (dashed line).

The blue gemstone par excellence is the *sapphire*, a variety of corundum—Al_2_O_3_. The mechanism of the blue colour generation in sapphires has long been debated [16]. Bristow et al. [17] provided spectroscopic evidence that the mechanism responsible is an intervalence charge transfer (IVCT) between Ti^4+^ and Fe^2+^; Palanza et al. [18,19], besides the IVCT mechanism, cited overlapping crystal field transitions of Cr^2+^, Cr^3+^, Ti^3+^, V^2+^ and V^3+^ ions. In Figure 2, the FORS spectrum of a blue sapphire is shown: it is characterised by main absorption bands at ca. 390, 456 and 706 nm due to Fe^3+^ and a band at 570 nm due to Fe^2+^-Ti^4+^ IVCT.

Sapphires, which usually are blue, can also be yellow–orange due to the presence of Fe^3+^. The corresponding spectrum shows the absorption band at ca. 450 nm more prominent and a shoulder at 413 nm (see Figure 2). Sapphires may also appear green when both yellow and blue chromophores are present.

Additional spectral features in sapphires can include two luminescence bands at 693 and 694 nm, due to Cr^3+^ ions [20,21], that appear as a single sharp negative band in the FORS spectrum (see Figure 1).

Recently [22,23], it has been demonstrated that UV-visible spectrophotometry, besides other techniques [24], can differentiate treated and non-treated corundum, which is an important issue in the gemstones market, based on the presence of a strong, wide absorption band at ca. 555 nm due to the formation of the blue colour [FeTi]^6+^ complex.

**Figure 3:**
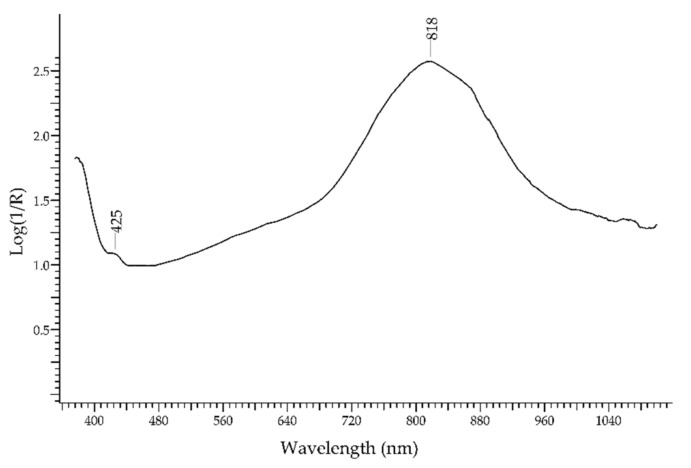
FORS spectrum in Log(1/R) coordinates of aquamarine.

The presence of Fe^2+^ and Fe^3+^ ions, instead, causes the typical blue–green colour of *aquamarine*, which is due to a variety of beryl—Be_3_Al_2_Si_6_O_18_. The spectrum (Figure 3) shows two main absorption bands at 425 nm, due to Fe^3+^ in octahedral sites, and at ca. 820 nm, due to Fe^2+^-Fe^3+^ intervalence charge transfer [25].

**Figure 4:**
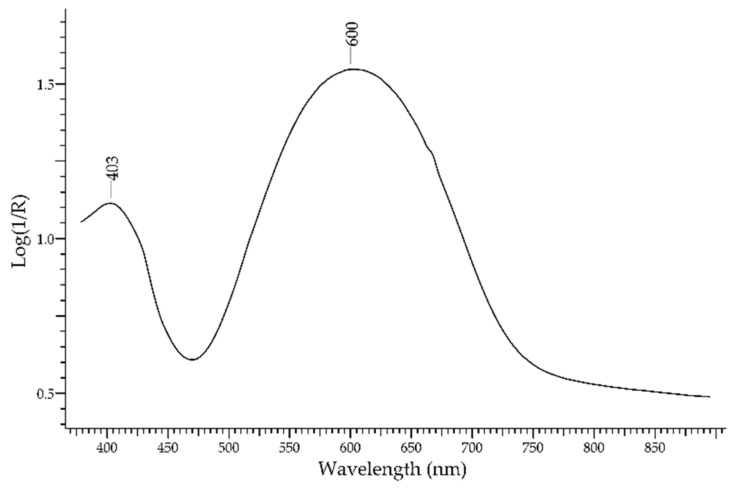
FORS spectrum in Log(1/R) coordinates of lapis lazuli.

Another precious blue stone, known by mankind since at least 5 millennia, is the *lapis lazuli*. The typical blue colour is due to the lazurite phase—Na_6_Ca_2_(Al_6_Si_6_O_24_)(SO_4_,S,S_2_,S_3_,Cl,OH)_2_—while other accessory phases (e.g., diopside, calcite, pyrite) are present but do not contribute significantly to the absorption features. The FORS spectrum (Figure 4) is dominated by a main band at 600 nm and a second band at ca. 400 nm, which are both due to the intervalence charge transfer mechanism of absorption between HS_3_^−^, S_2_^−^ and S_3_^−^ radicals entrapped in the lazurite cage. The second band has been considered as distinctive for samples of Afghan origin [26].

**Figure 5:**
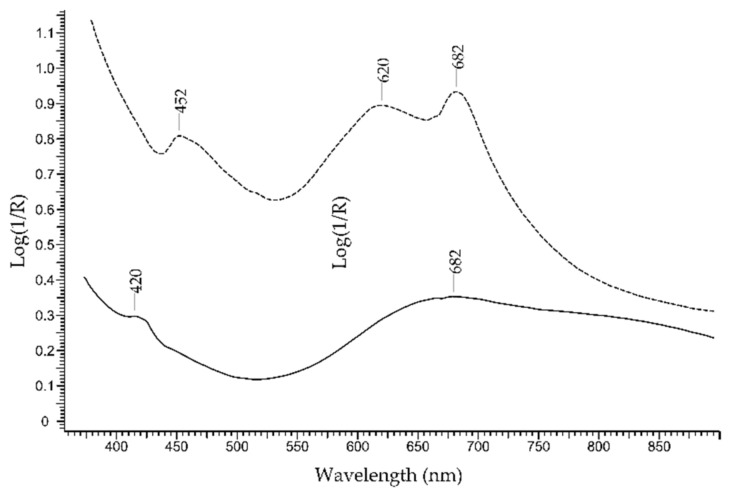
FORS spectra in Log(1/R) coordinates of a natural turquoise (solid line) and a dyed turquoise (dashed line).

The *turquoise* is considered a step below the most precious gemstones. It is a hydrated Cu and Al phosphate with formula CuAl_6_(PO_4_)_4_(OH)_8_·4H_2_O. The colour is mainly due to Cu2+, with contribution by substituted elements such as Fe^2+^, Fe^3+^ and Zn^2+^. The FORS spectrum of turquoise (Figure 5) shows a broad band at ca. 680 nm due to Cu^2+^ and a sharp peak at ca. 420 nm due to Fe^3+^, accounting for a greener hue [27]. The presence of broad absorption bands at ca. 620 and 680 nm usually indicates dyed turquoises [28].

### 2.2. Green Gemstones

**Figure 6:**
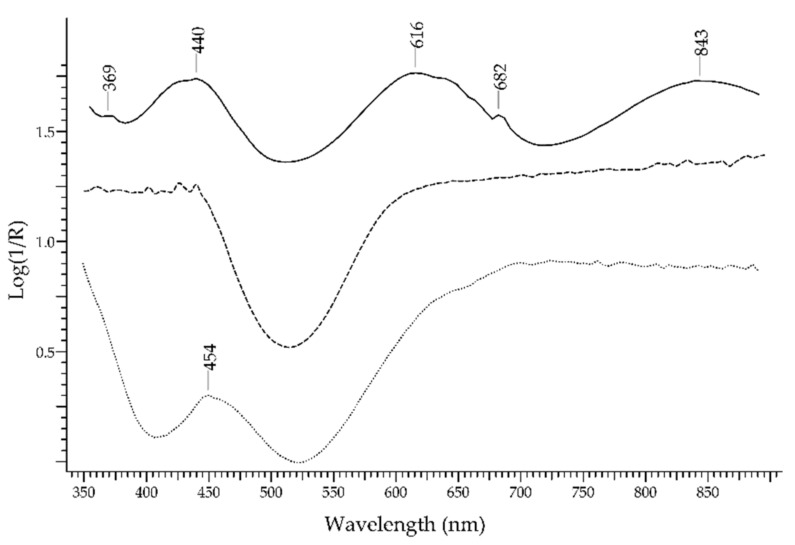
FORS spectra in Log(1/R) coordinates of an emerald (solid line), a Cu^2+^ green glass (dashed line) and a Cu^2+^/Cr^3+^ green glass (dotted line).

The most important green gemstone is the *emerald*, which is the most precious variety of beryl—Be_3_Al_2_(Si_6_O_18_). The UV-visible absorption spectrum of emerald obtained by FORS is well known and characterised by two main broad bands occurring at 440 and 616 nm, due to Cr^3+^ and V^3+^ ions, and two sharp bands occurring around 682 nm, due to Cr^3+^ ions [29]. Spectral features due to Fe^2+^ (a broad band at 843 nm) and Fe^3+^ ions can be present as well; the presence or absence of these features can provide information on the geographic provenance of emeralds. In Figure 6, the spectrum of an emerald is shown in comparison with the spectra of an emerald-like green glass coloured with Cu^2+^ and of an emerald-like green glass coloured with Cr^3+^and Cu^2+^ (Kremer Pigmente 39132, Colored Glass, Emerald Green, transparent): the possibility of distinguishing emerald from the two glasses according to the absorption features is apparent, even in the case of glass containing Cr^3+^, which is the same ion that generates the colour of emerald but inside a ligand field of different strength.

**Figure 7:**
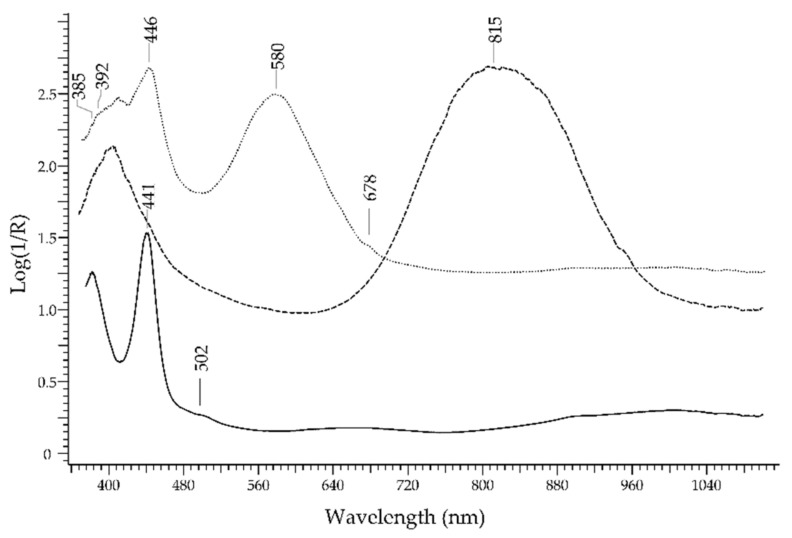
FORS spectrum in Log(1/R) coordinates of alexandrite (dotted line), a green heliodor (dashed line) and a yellow chrysoberyl (solid line).

Another gemstone containing beryllium is the *alexandrite*, which is a variety of chrysoberyl—BeAl_2_O_4_. It is a rare pleochroic gemstone that appears green in daylight but turns red in incandescent light [30]. The colour is due to impurities of the Cr^3+^ ion substituting Al^3+^ in the structure, which generate a main band at ca. 580 nm and a shoulder at ca. 680 nm; another band at 446 and minor bands between 380 and 400 nm are due to Fe^3+^. Figure 7 shows a comparison of the spectra of alexandrite, a green heliodor (another gemstone of the beryl family) and of a yellow chrysoberyl (see below).

**Figure 8:**
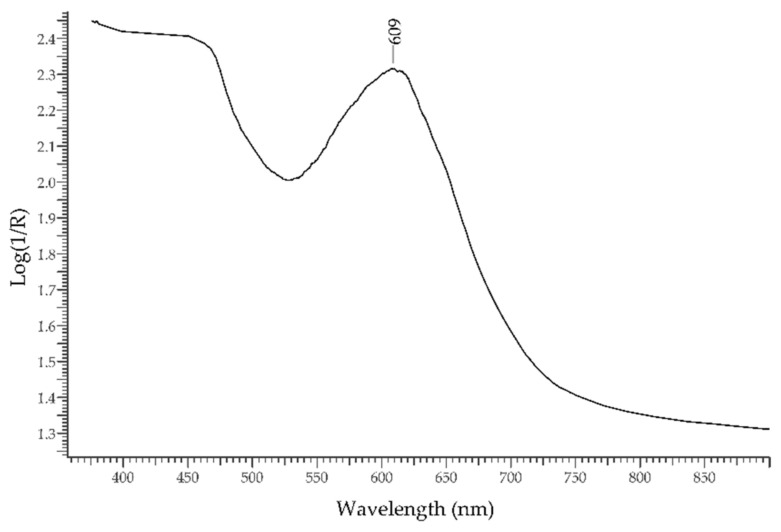
FORS spectrum in Log(1/R) coordinates of chrome-chalcedony.

The *chrome-chalcedony* or *mtorolite* is a rare variety of chalcedony. Its aspect is green due to Cr^3+^ impurities, and its reflectance spectrum (Figure 8) is dominated by a single band at ca. 610 nm. It was commonly employed in glyptic art of the Roman age [31], possibly as a substitute of emerald, and in medieval precious bindings such as the *Pace di Chiavenna* [32] and the binding of the C Codex of Vercelli [33].

A very common gemstone is the *malachite*. Its absorption spectrum is dominated by a peak at ca. 800 nm due to Cu^2+^ [34]; because this gemstone is opaque, it can be distinguished from a transparent green glass but not from an opaque green glass coloured with Cu^2+^, which will show similar absorption features. *Chrysocolla* is coloured by Cu^2+^ too, but the absorption maximum occurs at ca. 690 nm [35].

**Figure 9:**
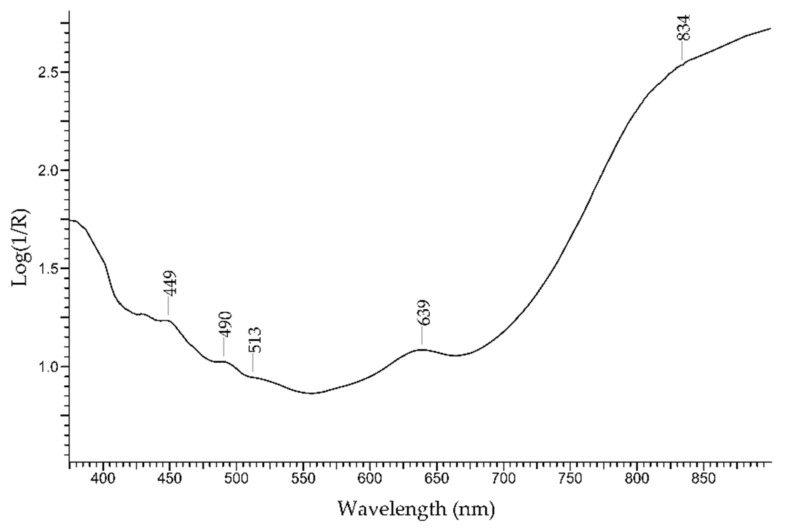
FORS spectrum in Log(1/R) coordinates of peridot.

The *peridot* is a green-to-yellow variety of the mineral olivine, with formula Mg_2_SiO_4_·Fe_2_SiO_4_. (Figure 9). The colour is due to Fe^2+^ ion [36] (weak bands between ca. 450 and 490 nm); other features at 513 and 653 nm can possibly be due to another chromophore such as Cr^3+^.

### 2.3. Pink Gemstones

**Figure 10:**
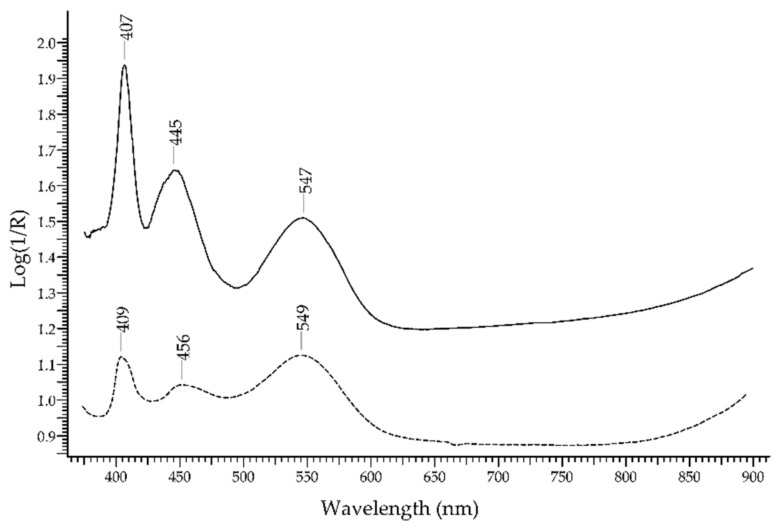
FORS spectra in Log(1/R) coordinates of rhodochrosite (solid line) and rhodonite (dashed line).

The *rhodochrosite*, a Mn(II) carbonate with formula MnCO_3_, is one of the pink gemstones. The colour is due to the three bands of Mn^2+^ occurring at 407, 445 and 547 nm (Figure 1). Another Mn-containing pink gemstone is the *rhodonite*, a silicate with formula MnSiO_3_. The spectral features are very similar to those of rhodochrosite, with Mn^2+^ bands occurring at 409, 456 and 549 nm (Figure 10). Therefore, FORS systems with the 350–1100 nm spectral range will generally be unable to distinguish between rhodochrosite and rhodonite, while systems with an extended range up to 2500 nm will reveal spectral features of the anions (carbonate and silicate in this case), thus enabling a more accurate identification.

### 2.4. Red and Purple Gemstones

Ruby, the most precious variety of corundum—Al_2_O_3_—owes its colour to Cr^3+^ [16]. It is relatively easy to identify and discriminate from its substitutes, such as ruby-like glasses, thanks to two luminescence bands occurring at 693 and 694 nm, due to Cr^3+^; these bands can be clearly seen upon reflectance measurements, despite not being true reflectance bands.

**Figure 11:**
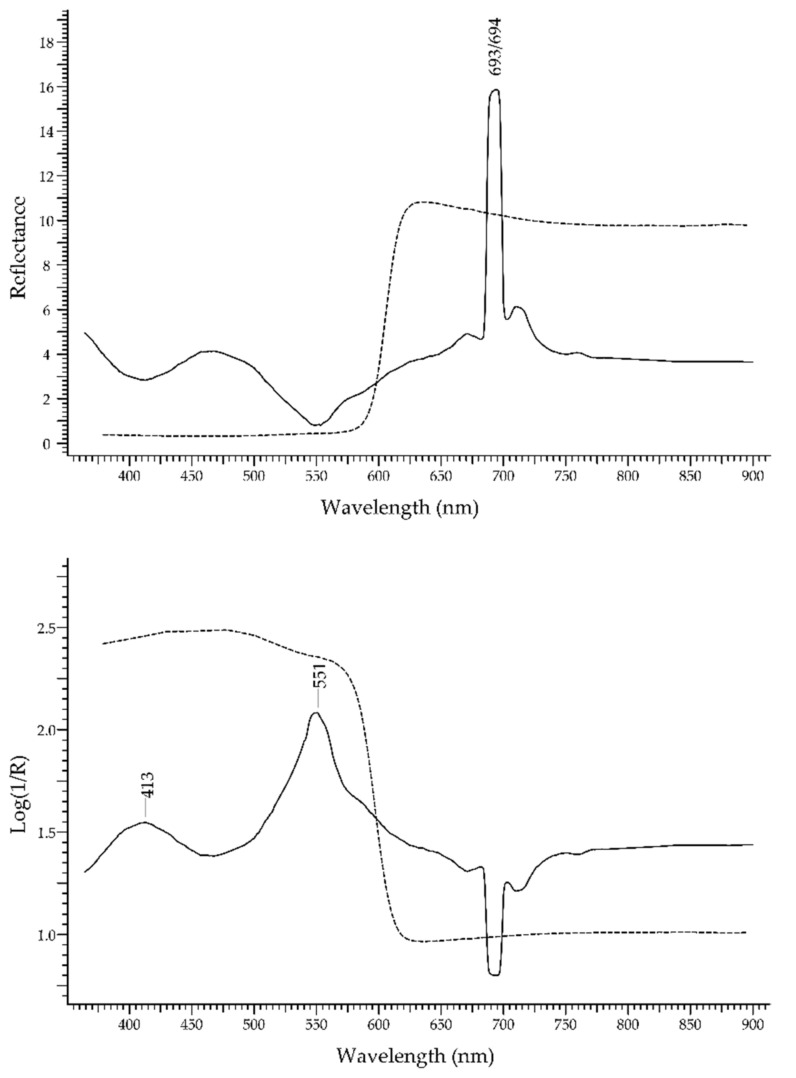
**Top**: FORS spectra in reflectance coordinates of ruby (solid line) and a ruby-coloured glass (dashed line). **Bottom**: FORS spectra in Log(1/R) coordinates(same legend).

In Figure 11 (top), the FORS spectra of ruby and a ruby-like glass coloured with selenium (Kremer Pigmente 39224, Colored glass, Gold ruby extra, transparent) are shown in reflectance coordinates: the spectrum of ruby (solid line) is clearly dominated by the two luminescence bands. In Log(1/R) coordinates (Figure 11, bottom), the spectrum of ruby shows two absorption bands at ca. 413 and 550 nm, which were again due to Cr^3+^.

The difference between the spectral features of ruby and a ruby glass are evident. In the former, the chromophore system is the Cr^3+^ ion in the network of corundum (Al_2_O_3_) which causes the crystal-field splitting of the energy levels of Cr^3+^ [37]. In the latter, the ruby colour can be obtained by adding Se, metallic Cu or metallic Au, but whatever the chromophore is, the spectral features are widely different from those of Cr^3+^ ion in corundum.

**Figure 12:**
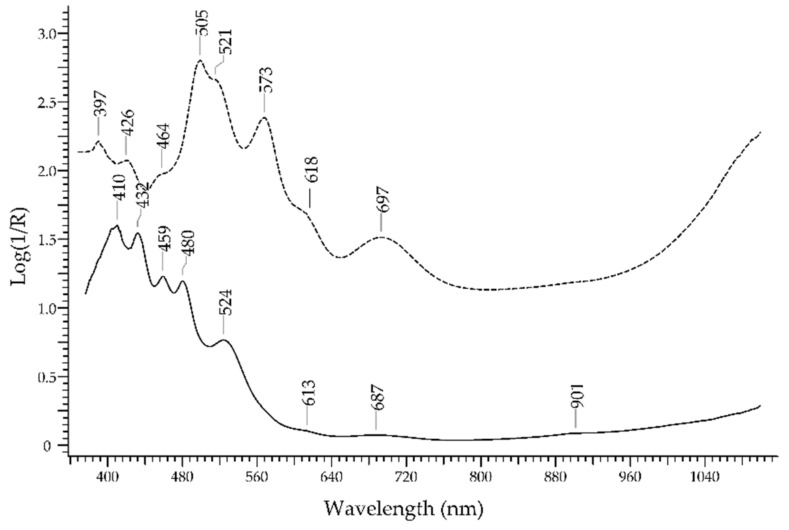
FORS spectrum in Log(1/R) coordinates of a spessartite garnet (solid line) and a purple pyrope–almandine garnet (dashed line).

The garnet group of minerals has a broad range of chemical composition and colours from the most common red to orange, purple, brown, up to colourless [38]. The spectrum of garnet is depending on the transition metal ions present in the structure, mostly Cr^3+^, Fe^2+^, Fe^3+^, Mn^2+^ and V^3+^ [39,40] and on Fe^2+^-Ti^4+^ intervalence charge transfer, and it is usually rich in features. In the spectrum in Figure 12, taken from a purple pyrope-almandine garnet (dashed line), absorption bands at 397, 464, 505, 521, 618 and 697 nm can be attributed to Fe^2+^, with the last band attributable also to Fe^3+^-Fe^2+^ IVCT; bands at 426 and 464 nm can be attributed to Mn^2+^. The band at 573 nm may be attributed to Fe^3+^, Cr^3+^ and/or V^3+^. The spectrum of an orange spessartite (solid line) is dominated by the features of Mn^2+^. at 410, 432 and 480 nm.

**Figure 13:**
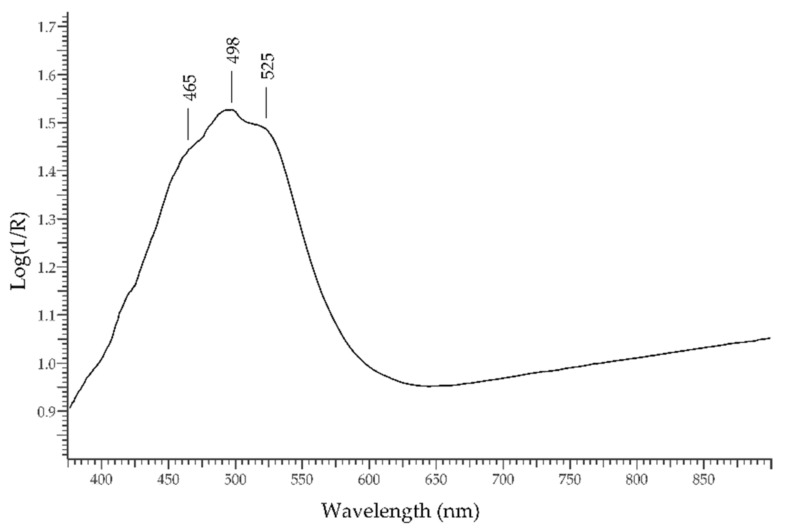
FORS spectrum in Log(1/R) coordinates of a natural coral.

A particular case of red gemstone is the *coral*, one among the very few of organic nature. The red-to-pink colour of corals is not due to inorganic chromophores but to carotenoids; therefore, it is produced by electronic transitions among delocalised molecular orbitals [41]. Natural corals can be counterfeit by bleaching and dyeing the surface in order to obtain a more homogeneous and rich coloration. In natural corals, the absorption spectrum is dominated by a main band structured in three sub-bands at ca. 465, 498, and 525 nm, with minor spectral features in the UV region. Dyed coral samples do not show these features. In Figure 13, the FORS spectrum of a natural coral is shown.

### 2.5. Violet Gemstones

**Figure 14:**
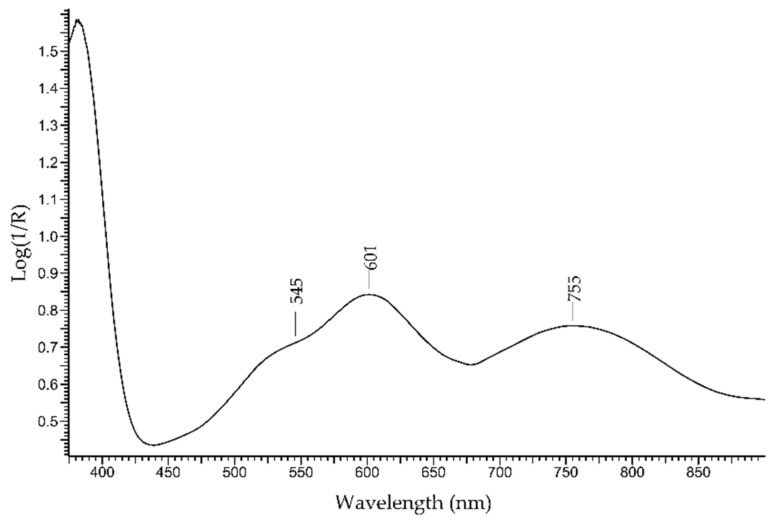
FORS spectrum in Log(1/R) coordinates of tanzanite.

A violet–blue gemstone is the *tanzanite*, which is a pleochroic variety of zoisite—(Ca_2_Al_3_[Si_2_O_7_][SiO_4_]O(OH)). The colour is mainly due to the V^3+^ ion [42]. Due to the rarity of natural high-quality gemstones, lower-quality products are generated by means of heat treatment. The absorption spectrum is dominated by the features of V^3+^ with two main bands occurring at 600 and 750 nm and a shoulder at 540 nm (Figure 14).

### 2.6. Yellow Gemstones

The *chrysoberyl* is a gemstone containing beryllium as the beryl family, of various colours, although yellow and yellow–green are considered the most valuable. The FORS spectrum is dominated by a sharp band at ca. 440 nm due to Fe^3+^, with a minor band at 502 nm (Figure 7).

Heliodor is another variety of beryl with golden–green to yellow–green hue. Its colour is due to a mechanism of charge transfer between Fe^3+^ ions and the surrounding oxygen ions [43], generating an absorption band at ca. 815 nm (Figure 7).

### 2.7. Multicoloured Gemstones

These gemstones represent a challenge for FORS analysis. In such gemstones, the presence of metal ions impurities or other mechanisms of colour generation can vary extensively, rendering the absorption response highly variable (although not the vibrational behaviour), and it is difficult to identify specific spectral features. In some cases, the same gemstone may include areas with different colours [44].

A very common group of multicoloured gemstones is that of *quartz*—crystalline SiO_2_. It includes macro-, micro- and crypto-crystalline varieties, with a wide range of colours arising from colour centres, from optica effects and from inclusions. [45]. The set of varieties has recently been reviewed by Jovanovski et al. [46]. As to the most valuable quartz gemstones, that is the macro-crystalline varieties, the main mechanism generating colour is that of colour centres associated with ions external to the structure of quartz, mostly Fe^3+^ and Al^3+^ [47]. *Amethyst*, the most precious variety of quartz, owes its violet colour to Fe^3+^ impurities exposed to ionising radiation, arising from the natural decay of ^40^K nuclides or from artificial irradiation; irradiation causes the oxidation of substitutional Fe^3+^ to Fe^4+^ and reduction in interstitial Fe^3+^ to Fe^2+^. [48]. Its FORS spectrum (Figure 1) is characterised by a broad absorption band at ca. 540 nm; further bands are present at ca. 350 and 950 nm. It is not possible to distinguish between naturally or artificially irradiated amethysts by means of the FORS response only.

**Figure 15:**
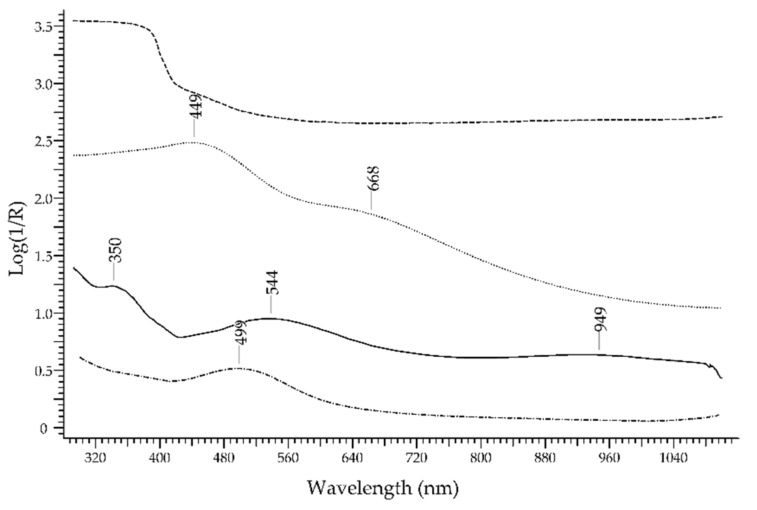
FORS spectrum in Log(1/R) coordinates of amethyst (solid line), citrine quartz (dashed line), smoky quartz (dotted line) and rose quartz (dashed–dotted line).

Another variety is *citrine quartz*, with a yellow to brown colour. While natural citrines are rare, most of them are obtained by heat treatment of amethysts between 350 and 450 °C: this will increase the number of substitutional and interstitial sites filled with Fe^3+^. The FORS spectrum (Figure 15) has no specific features, showing only a generic decrease towards NIR.

*Smoky quartz* is a variety in which the colour centres, created by natural or artificial irradiation, are associated with impurities of Al^3+^. The FORS spectrum (Figure 15) will present a main band at ca. 450 nm with a shoulder at ca. 670 nm. Additional colours can be generated by heating, either natural or artificial.

Finally, *rose quartz*, the well-known variety of pink colour, owes its chromatic features to fibrous inclusions of dumortierite and in particular to IVCT between Fe^2+^ and Ti^4+^ that occur as impurities inside this aluminoborosilicate mineral [49]. The FORS spectrum (Figure 15) contains a main band centred at ca. 500 nm and assigned to the above-mentioned IVCT.

**Figure 16:**
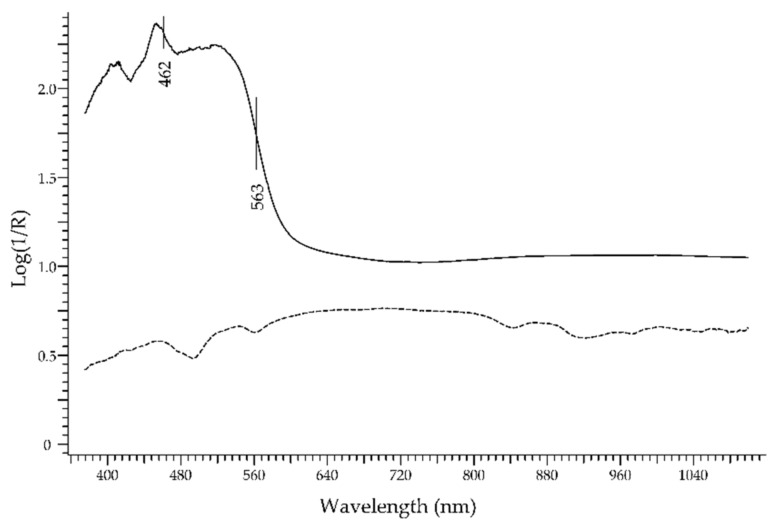
FORS spectrum in Log(1/R) coordinates of a Mexican fire opal (solid line) and an Ethiopian dyed opal (dashed line).

The *opal* is an unusual gemstone. It is a hydrous silica material—SiO_2_·nH_2_O—with different degrees of crystallinity and crystal structure. It can be colourless, white, yellow, orange, or red, besides other minor colour varieties. The particular aspect of precious opals, also called *noble opals*, is due to the diffraction of light by the regular stacking of the silica microspheres forming the body of the gemstone. The colour can be caused by specific mineral phases; red–orange hues are usually associated with iron oxides. Hydrophane gemstones from Ethiopia, which are very porous and can thus easily absorb water and be subjected to dyeing or impregnation processes, can result in opals with an aspect similar to fire opals from Mexico [50]. Wu et al. [51] have recently studied the possibility to distinguish natural fire opals from dyed opals. Figure 16 shows a natural fire opal from Mexico (solid line), with two inflection points at 462 and 563 nm that can be assigned to hydrated iron oxides rather than to hematite [34], and an Ethiopian dyed opal (dashed line) with features due to the impregnating solution.

**Figure 17:**
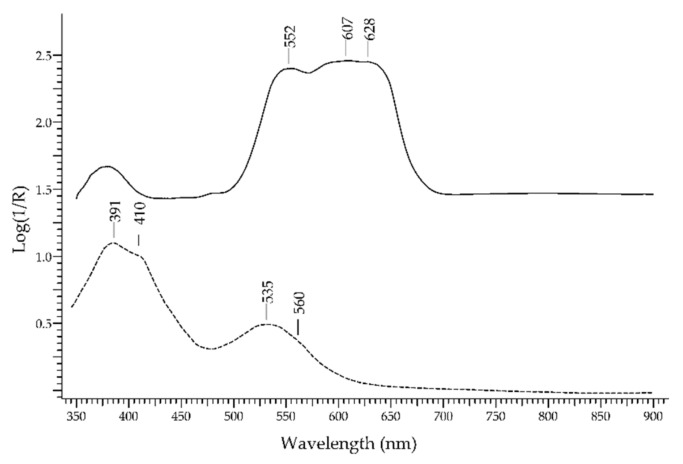
FORS spectrum in Log(1/R) coordinates of a red spinel (solid line) and a blue spinel (dashed line).

The family of *spinel* comprises different members that can be defined as multiple oxides with a highly variable composition [52]. The simplest composition is AB_2_O_4_ with A representing a divalent ion and B representing a trivalent ion. This structure can accommodate different transition element ions that act as chromophores, such as Co^2+^, Cr^3+^, Cu^2+^, Fe^2+^, Fe^3+^, Mn^2+^, Mn^3+^, and V^3+^. Consequently, there is a wide range of displayed colours due to diverse absorption features determined by the presence of different transition elements. The most common spinels are red, coloured by Cr^3+^; blue, coloured by Fe^2+^ or Co^2+^; and pink, coloured by both Cr^3+^ and Fe^2+^. Figure 17 shows two examples of spectra. In the spectrum of a red spinel, the band at 390 and the shoulder at 560 nm are attributed to V^3+^, while the bands at ca. 410 and 535 nm are attributed to Cr^3+^. In the spectrum of a blue spinel, the typical spectral signature of Co^2+^ in the tetrahedral site can be detected.

**Figure 18:**
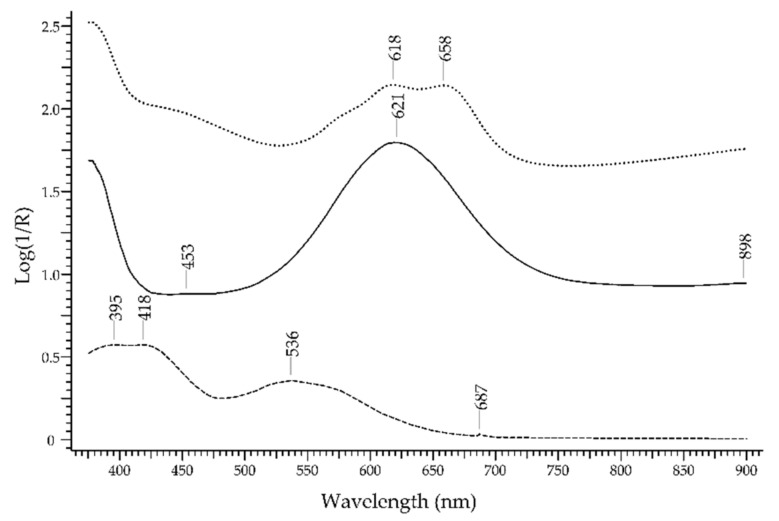
FORS spectrum in Log(1/R) coordinates of a blue topaz (solid line), a pink natural topaz (dashed line) and a green irradiated topaz (dotted line).

The *topaz* is a particularly challenging case for the FORS technique. Its formula is Al_2_SiO_4_(F,OH)_2_, but its colour can vary from blue to green, yellow, pink, brown, and it can even be colourless. In addition, it is a *pleochroic* gemstone. Blue is the most common colour of topaz on the market, but natural topazes are rarely blue; indeed, the hue is obtained artificially by means of heat treatment and irradiation. The spectrum shows a main band at 620–650 nm depending on the measurement angle (Figure 18), which is possibly due to irradiation-induced defects [53] or to Cr^3+^, Fe^2+^ and Mn^2+^ ions [54]. A pink colour characterises the so-called *imperial topaz* but is instead caused by Cr^3+^ ion according to the bands at 395, 418, 536 and 687 nm. Green-irradiated topaz shows features at 618 and 658 nm.

**Figure 19:**
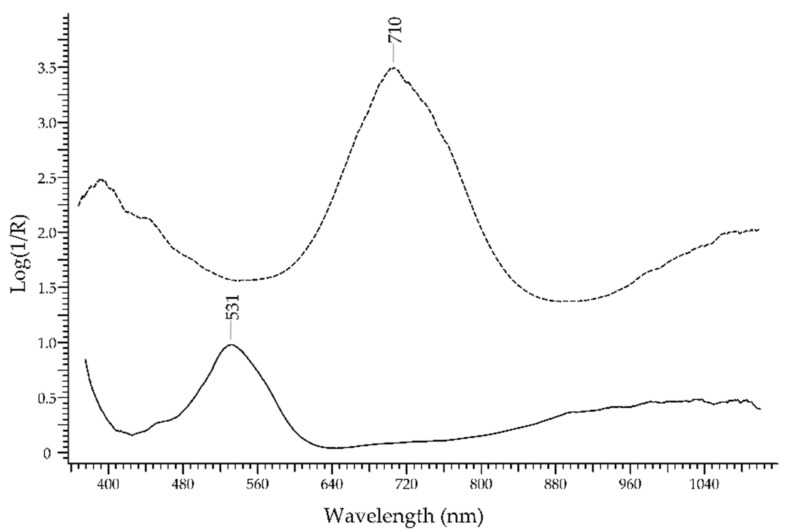
FORS spectrum in Log(1/R) coordinates of a rubellite variety (solid line) and a green tourmaline (dashed line).

*Tourmaline* is the name of a large group of gemstones that share a common crystal structure (hexagonal) but have different compositions. The basic formula is XY_3_Z_6_(T_6_O_18_)·(BO_3_)_3_V_3_W, with X, Y, Z, T, V, and W representing different elements and, hence, different chromophore systems are possible. The main ions generating colour are Cr^3+^, Cu^2+^, Fe^2+^, Fe^3+^, Mn^2+^, Mn^3+^, Ti^4+^ and V^3+^. The transition mechanisms can be due to the ligand field and/or to IVCT. Therefore, as in the case of topaz, the tourmaline group is highly challenging for the FORS technique, since it contains members of nearly all colours. Figure 19 shows a very limited example of the many varieties: a red *rubellite*, with its colour ascending from the absorption band at ca. 530 nm due to Mn^3+^ [55], and a green tourmaline with an intense absorption band at ca. 710 nm due to Fe^2+^ ion and Fe^2+^-Ti^4+^ IVCT.

**Figure 20:**
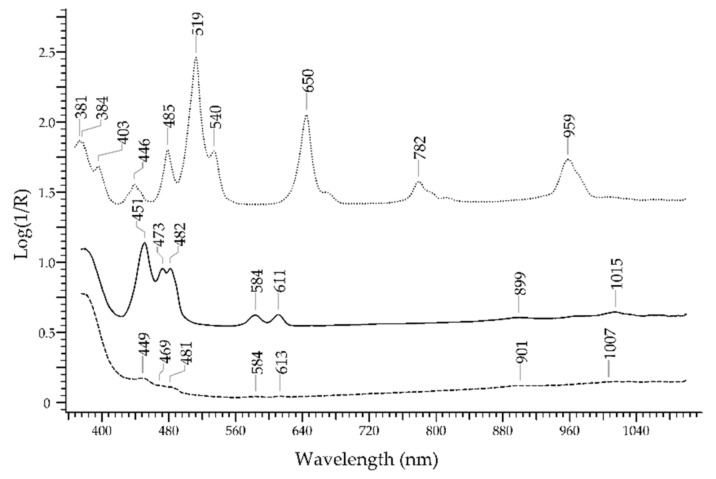
FORS spectrum in Log(1/R) coordinates of a green (solid line), a pink (dotted line) and a yellow (dashed line) zircon.

The *zircon* family comprises gemstones with the same formula—ZrSiO_4_—showing different colours according to the chromophores. The origin of the colour is not entirely clear [56]: pure zircon is colourless (it can a substitute of diamond), but more frequently, the natural content of U^4+^ or Th^4+^ ions substituting Zr^4+^ in the structure generates blue gemstones. The radioactive decay of these ions causes radiation damages that in turn generate colour centres and the increase in red–brown and amber colours. The absorption features of zircons can be highly variable; as an example, in Figure 20, the spectra of a green, a pink and a yellow zircon are shown. The features are mainly due to U^4+^ ion and to colour centres.

### 2.8. Uncoloured Gemstones

This group of gemstones constitutes a clear limit to the possibilities of FORS in their identification. Uncoloured transparent gemstones, such as *diamond* or *rock crystal* (uncoloured quartz), yield very poor—if any—spectral responses in the analysis in reflectance mode. Whether cut gemstones or rough stones, the exciting light enters the material, undergoes several refractions inside it and does not exit or exits very faintly; the result is a nearly flat line at 0% reflectance. The well-known N3 centre of diamonds, a lattice defect constituted by 3 nitrogen atoms bonded to a vacancy, causes an absorption band at 415.2 nm, but this cannot be seen by FORS. As a comparison, Drift-FT-IR spectroscopy can differentiate diamond from cubic zirconia and synthetic moissanite which resemble it [57]. Lipatov et al. [58] claimed that optical absorption spectroscopy combined with cathodoluminescence spectroscopy can be used for identifying natural and synthetic diamond, but they exploited spectra obtained in transmittance mode, not in reflectance.

### 2.9. Glassy Materials

Glasses and vitreous pastes were commonly used in medieval jewellery artworks, possibly as substitutes of authentic gemstones [33]. FORS analysis cannot highlight the glassy nature of a gemstone, of course, but it can provide indirect identification by yielding information on the chromophore system. This is particularly true as far as vitreous pastes are concerned, being them opaque materials. The main metal ions that impart colour to glass, i.e., Co^2+^, Cu^2+^, Fe^2+^ and Fe^3+^, Mn^3+^ and Mn^4+^, etc. can be identified in the FORS spectrum according to their typical absorption bands [59], therefore suggesting the presence of glassy gemstones.

### 2.10. Comparison of FORS with other Techniques

The diagnostic potential of FORS in the correct identification of gemstones has been tested by comparison of the FORS responses with those obtained with Raman spectroscopy and with refractometry in the analysis of three precious medieval bindings: the *Pace di Ariberto* o *Evangeliario di Ariberto*, held in the Museo del Tesoro del Duomo at Milan (Italy), the *Pace di Chiavenna*, held in the Museo del Tesoro di San Lorenzo at Chiavenna (Lumbardy, Italy) and the *Legatura di Vercelli*, held in the Museo del Tesoro del Duomo at Vercelli (Piedmont, Italy). These notable jewellery artworks are datable to the 11^th^ century and are decorated with rich and various gemstone goods. In particular, the gemstones on the *Pace di Ariberto* [60] and the *Legatura di Vercelli* [33] have been previously analysed with Raman spectroscopy, while the gemstones on the *Pace di Chiavenna* have been analysed with refractometry by an expert gemmologist [32]. Table 1 shows the results of the comparison.

It is apparent that the diagnostic performances of FORS are satisfying in all three cases: if pearls, rock crystals and glassy materials are not considered, between 88 and 96% of the identification of the gemstones is correct.

### 2.11. Chemometric Treatment of Data

Among the greatest advantages of the FORS technique is the speed of analysis: spectra can be collected in as low as 1 s, so that several spectra can be acquired in a short time. This justifies the fact that FORS can be proposed as a survey technique in the identification of gemstones on a complex jewellery artwork. After collecting several spectra, it can be useful to treat them with multivariate analysis in order to identify groups of gemstones with similar features. Using a well-known chemometric pattern recognition method, Hierarchical Cluster Analysis (HCA), it is possible to discriminate gemstones, colour by colour, according to their composition—or better to their chromophore system—which is reflected inside the FORS spectrum as minima or luminescence peaks. As an example, this approach is shown in the discrimination of the green gemstones contained in the three above cited medieval bindings. The gemstones were the following:

26 emeralds (em);
10 emerald-like glasses coloured with Cu^2+^ (gg em);
6 green glasses coloured with Ni^2+^ (gg);
2 chrome-chalcedony gemstones (cc).

**Figure 21:**
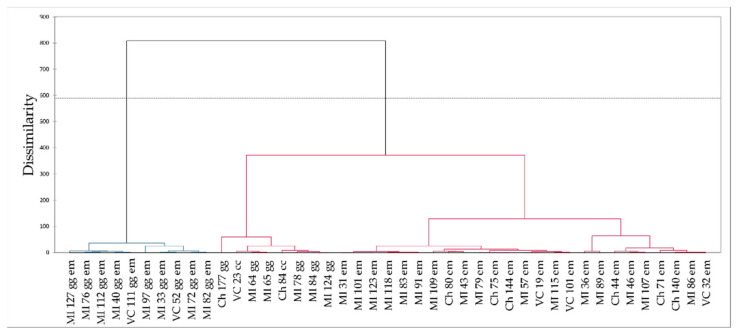
Dendrogram obtained by means of HCA on the FORS spectra of green gemstones.

A total of 44 green gemstones have been included in the analysis. The FORS spectra have been pre-treated by selecting the range 250–900 nm with a 1 nm path; this yielded 650 variables. Then, range scaling has been applied along the spectrum. After HCA, the dendrogram shown in Figure 21 was obtained. The result highlights the differences among the three main types of gemstones, arising from the spectral features of their FORS responses. The two chrome-chalcedony gemstones were classified among the group of green glasses.

## 3. Discussion

As it has been described in the previous paragraphs, one clear disadvantage of the FORS technique in the identification of gemstones is its strict dependence from the chromophore system and not from the structure of the gemstone. Nevertheless, apart from diamond, the most important gemstones, i.e., ruby, sapphire and emerald, can be easily discriminated from other gemstones with similar hues. This topic will be discussed in the next paragraphs.

### 3.1. Ruby vs. Red Gemstones

**Figure 22:**
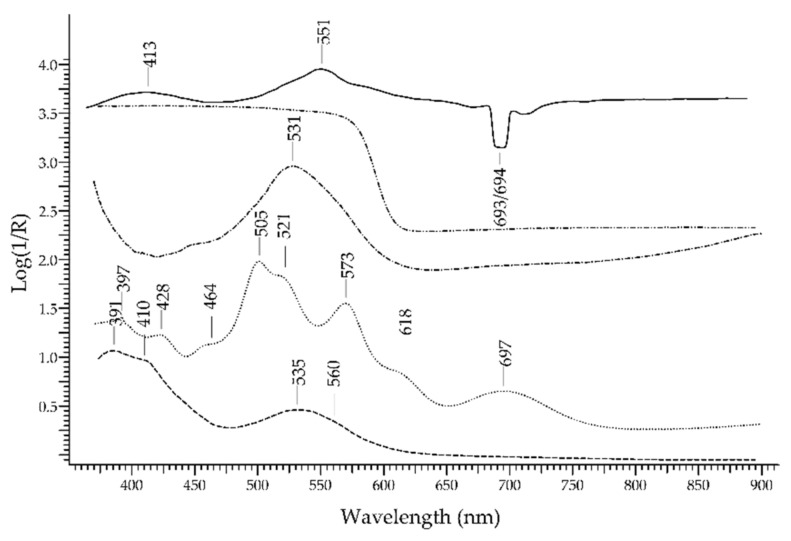
FORS spectrum in Log(1/R) coordinates of ruby (solid line), red spinel (dashed line), ruby garnet (dotted line), red tourmaline (dashed–dotted line) and a ruby-like glass (dashed–dotted–dotted line).

The possibility of discriminating ruby from red–purple gemstones (Figure 22) relies mostly on the typical luminescence bands of ruby, due to Cr^3+^, that do not even occur in glasses containing Cr^3+^. The two absorption bands occurring at 413 and 550 nm are not selective enough to allow a reliable identification. The red spinel, in fact, has a similar chromophore, i.e., Cr^3+^, which generates absorption bands at 410 and 535 nm in addition to a band at 390 nm due to V^3+^; however, the spectrum of red spinel generally lacks the strong luminescence bands at 693/694 nm. Purple garnets, though showing a somewhat similar hue, have a totally different spectral fingerprint with several bands (397, 426, 464, 505, 521, 573, 618 and 697 nm) due to Cr^3+^, Fe^2+^, Fe^3+^, Mn^2+^ and V^3+^. The red tourmaline variety called rubellite shows a single absorption band at 530 nm due to Mn^3+^. Finally, ruby glasses, regardless of the chromophore, have generally sigmoid-like spectra.

### 3.2. Sapphire vs. Blue Gemstones

**Figure 23:**
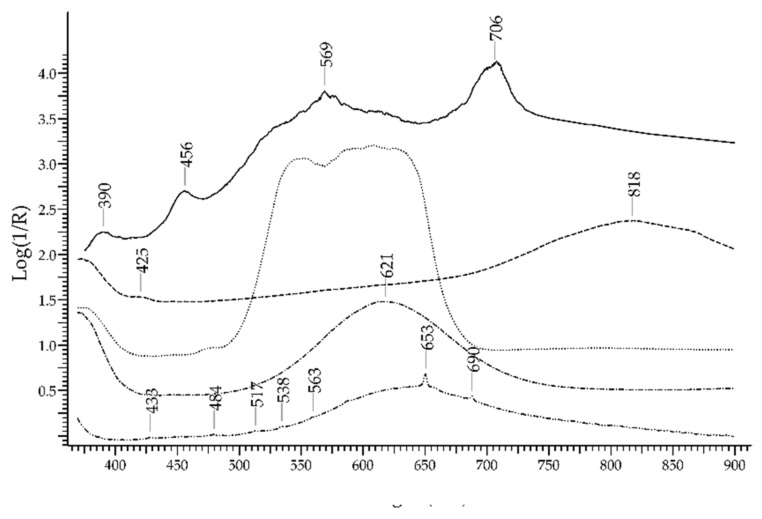
FORS spectrum in Log(1/R) coordinates of sapphire (solid line), a blue aquamarine (dashed line), a blue spinel (dotted line), a blue topaz (dashed–dotted line) and a blue zircon (dashed–dotted–dotted line).

The blue sapphire can be easily discriminated from blue aquamarine, blue spinel, blue topaz and blue zircon (lapis lazuli and turquoise are of course not considered, being opaque gemstones). The spectral features of sapphire, i.e., the bands at 390, 456 and 706 nm due to Fe^3+^ and the band at 570 nm due to Fe^2+^-Ti^4+^ IVCT, are selective enough to allow a reliable identification (Figure 23). Aquamarine, despite having Fe^2+^ and Fe^3+^ as the ions generating colour, shows a main band at ca. 820 nm. A blue spinel coloured by Co^2+^ will show the typical signature of the ion with three sub-bands between 550 and 650 nm, while a blue spinel coloured by Fe^2+^ will show bands at 459, 655 and 902 nm. The blue topaz shows a main large band at ca. 620 nm, due to irradiation-induced defects or to Cr^3+^, Fe^2+^ and Mn^2+^ ions. The blue zircon has a complex spectrum with sharp bands due to U^4+^, so it can be easily recognised. Of course, blue glasses coloured with Co^2+^, Cu^2+^ or Fe^2+^ have spectral features quite different from those of sapphire.

### 3.3. Emerald vs. Green Gemstones

**Figure 24:**
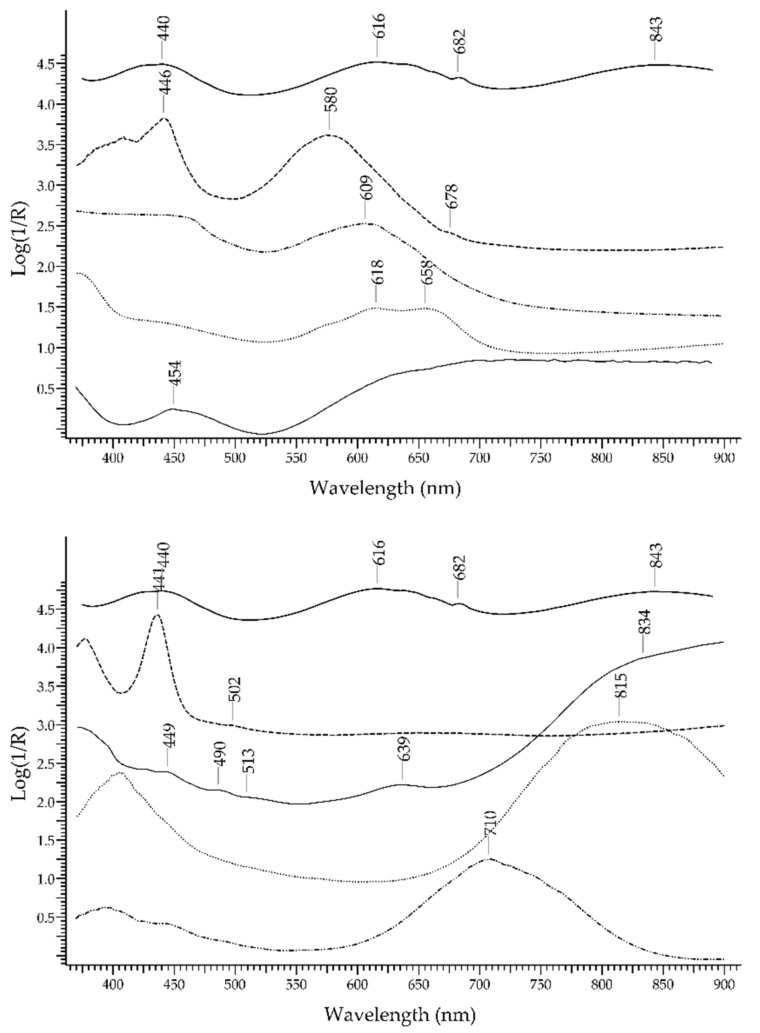
Top: FORS spectrum in Log(1/R) coordinates of emerald (solid line), alexandrite (dashed line), a green topaz (dotted line), a Cr^3+^ containing green glass (dashed–dotted line) and a chrome–chalcedony (dashed–dotted–dotted line). Bottom: FORS spectrum in Log(1/R) coordinates of emerald (solid line), chrysoberyl (dashed line), heliodor (dotted line), peridot (dashed–dotted line) and a green tourmaline (dashed–dotted–dotted line).

The discrimination between emerald and other green gemstones can be easily obtained despite the fact that some of the potential substitutes have a similar chromophore, i.e., the Cr^3+^ ion (Figure 24, top). The characteristic spectral features of emerald are two main bands occurring at 440 and 616 nm and two sharp bands occurring at 682 nm, which are all due to Cr^3+^ ion. Alexandrite, which has the same chromophore, shows a main band at ca. 580 nm and sharp bands at 682 nm, plus another band at 446 due to Fe^3+^. Chrome-chalcedony has a main band at 610 nm due to Cr^3+^ ion. Among the potential substitutes with Fe^2+^/Fe^3+^ chromophores (Figure 24, bottom), heliodor has a single band at 815 nm; chrysoberyl has a sharp band at ca. 440 nm, due to Fe^3+^, and a minor band at 502 nm; peridot shows only weak bands between 450 and 490 nm, due to Fe^2+^, and between 513 and 653 nm, due to Cr^3+^; green tourmaline has a main intense band at ca. 710 nm, due to Fe^2+^ ion and Fe^2+^-Ti^4+^ IVCT. Green topaz, a version obtained by irradiation, shows two bands at 618 and 658 nm. Finally, green glasses can be obtained by adding Cr^3+^, Cu^2+^, Ni^2+^ or V^3+^/V^5+^ [59], but in no case, even in that of a Cr^3+^-containing glass, is the resulting spectrum comparable to the one of emerald.

### 3.4. Final Considerations

The results described above show that the FORS technique have clear limits in the identification of gemstones but also clear advantages. A large number of gemstones can be identified; in cases where a gemstone has different varieties (i.e., topaz, tourmaline, quartz, etc.), the availability of a proper database can counteract the relative low diagnostic power of the technique.

The main advantage of the technique lies in its ease and speediness of use, that allows analysing jewellery artworks in a very safe way, without need of moving them outside their natural locations. The building of complete databases, that include the largest number of varieties, is a prerequisite for a proper use of this technique. In this view, apart from accessing databases available in the literature and on the web [61], the best choice is to build your own spectral database that is fully compatible with your own instrumental setup.

## 4. Materials and Methods

### 4.1. Samples of Gemstones

The gemstones analysed in this work, listed in Table 2, were provided by Effeffe Preziosi di Gilberto Faccaro & C. at Valenza (city, Italy). Their identification was confirmed by means of Raman spectroscopy.

### 4.2. UV-Visible Diffuse Reflectance Spectrophotometry with Optic Fibres (FORS)

FORS analysis was carried out with two instruments. Most of the measurements were performed with an Avantes (Apeldoorn, The Netherlands) AvaSpec-ULS2048XL-USB2 model spectrophotometer and an AvaLight-HAL-S-IND tungsten halogen light source; the detector and light source are connected with fibre optic cables to a 1.5 mm diameter FCR-7UV200-2-1,5 × 100 probe, which contains cables for both illumination and detection; therefore, incident and detecting angles were respectively 45° and −45° from the surface normal in order to exclude specular reflectance. The spectral range of the detector was 200–1160 nm; considering the range of emission of the light source, the optimal range of acquisition of spectra was 350–1100 nm. The best spectral resolution of the system, calculated as FWHM, was 2.4 nm. Diffuse reflectance spectra of the samples were referenced against the WS-2 reference tile, guaranteed to be reflective at 98% or more in the spectral range investigated. The investigated area on the sample was 1 mm diameter. In all measurements, the distance between probe and sample was 2 mm. The instrumental parameters were as follows: 10 ms integration time, 100 scans for a total acquisition time of 1 s for each spectrum. The reproducibility of the system, as far as the conditions (distance between probe and sample, angle of the probe) are kept constant, is better than 5% in terms of band position and height. The system was managed by means of AvaSoft 8 software running under Windows 10™.

### 4.3. Raman Spectroscopy

In order to confirm the identification of the gemstones analysed in this work, all of them were previously subjected to Raman analysis. For this task, a high-resolution dispersive Horiba (Villeneuve d’Ascq, France) LabRAM HR Evolution model spectrometer coupled with a confocal microscope was used. The instrument was equipped with 532, 633 and 785 nm excitation lasers, an 1800 lines/mm dispersive grating, an 800 mm focal length achromatic flat field monochromator and a multichannel air-cooled CCD detector. The spectral resolution was 2 cm^−1^. Spectra were taken with long working distance 50x and 80x objectives. All spectra were recorder at full laser power. Exposure time was 1–10 s according to needs (3 accumulations). The system was managed with LabSpec 6 software running under Windows 10™.

## Figures and Tables

**Table 1:** Comparison of the results obtained with FORS, Raman spectroscopy and refractometry (Ref) in the analysis of three precious bindings.

Gemstones	*Pace di Ariberto*		*Pace di Chiavenna*		*Legatura di Vercelli*	
	FORS	Raman	FORS	Ref	FORS	Raman
agate	-	1				
amethyst	24	24	6	6	5	5
carnelian	-	1				
chalcedony	-	2				
doublet	-	4				
emerald	16	16	7	7	3	3
garnet	11	11	55	56	14	14
glass/vitreous paste	-	23	-	2	-	31
mtorolite			-	1	-	1
pearl	-	21	-	93	-	23
rock crystal	-	18			-	2
sapphire	10	10	19	19	5	5
turquoise	2	2				
other stones		1		6		
total identified	63	134	87	190	27	84
unidentified by FORS	71		103		57	
total excluding pearls, rock crystals and glassy materials	63	72	87	95	27	28
unidentified by FORS excluding pearls, rock crystals and glassy materials	9		8		1	

**Table 2:** List of gemstones analysed in this work.

Gemstone	Provenance	Colour	Notes
alexandrite	Brazil	green	
amethyst	Brazil	violet	
aquamarine	Brazil	blue	
chrome-chalcedony	unknown	green	^1^
chrysoberyl	Brazil	yellow	
citrine quartz	Brazil	yellow	
coral	Italy	red	
emerald	Colombia	green	
garnet	India	purple	Pyrope–almandine
garnet	Kenya	orange	spessartite
glass with Cr^3+^		green	Artificial ^2^
glass with Se		red	Artificial ^2^
heliodor	Brazil	green-yellow	
lapis lazuli	Afghanistan	blue	
opal	Mexico	various	
opal	Ethiopia	various	
peridot	Sri Lanka	green	
rhodochrosite	Romania	pink	
rhodonite	Tanzania	pink	
rose quartz	Brazil	pink	
ruby	Myanmar	red	
sapphire	Cambodia	blue	
sapphire		yellow	artificial
smoky quartz	Brazil	grey	
spinel	Russia	blue	
spinel	Myanmar	red	
tanzanite	Tanzania	violet	
topaz	Brazil	blue	
topaz		green	artificial
topaz	Brazil	pink	
tourmaline	Brazil	green	
tourmaline	Brazil	red	rubellite
turquoise	China	turquoise	
turquoise	China	turquoise	dyed
zircon	Myanmar	blue	
zircon		green	artificial
zircon		pink	artificial
zircon		yellow	artificial

^1^ From the binding of the *C Codex* of Vercelli [33]. ^2^ Kremer Pigmente.

## Data Availability

The spectral database generated in this study will be soon rendered available on a dedicated web site.

## Sample Availability

Samples of the compounds are not available from the authors.
